# Genomics‐Driven Monitoring of *Fraxinus latifolia* (Oregon Ash) to Inform Conservation and EAB‐Resistance Breeding

**DOI:** 10.1111/mec.17640

**Published:** 2025-01-06

**Authors:** Anthony E. Melton, Trevor M. Faske, Richard A. Sniezko, Tim Thibault, Wyatt Williams, Thomas Parchman, Jill A. Hamilton

**Affiliations:** ^1^ Department of Ecosystem Science and Management Pennsylvania State University University Park Pennsylvania USA; ^2^ Southwest Biological Science Center United States Geological Survey Flagstaff Arizona USA; ^3^ Dorena Genetic Resource Center, USDA Forest Service Cottage Grove Oregon USA; ^4^ The Huntington San Marino California USA; ^5^ Forests Resources Division, Oregon Department of Forestry Salem Oregon USA; ^6^ Department of Biology University of Nevada Reno Reno Nevada USA

**Keywords:** conservation, *Fraxinus latifolia*, genomic offset, landscape genomics, Oregon ash, threatened species

## Abstract

Understanding the evolutionary processes underlying range‐wide genomic variation is critical to designing effective conservation and restoration strategies. Evaluating the influence of connectivity, demographic change and environmental adaptation for threatened species can be invaluable to proactive conservation of evolutionary potential. In this study, we assessed genomic variation across the range of *Fraxinus latifolia* , a foundational riparian tree native to western North America recently exposed to the invasive emerald ash borer ( *Agrilus planipennis* ; EAB). Over 1000 individuals from 61 populations were sequenced using reduced representation (ddRAD‐seq) across the species' range. Strong population structure was evident along a latitudinal gradient, with population connectivity largely maintained along central valley river systems, and a centre of genetic diversity coinciding with major river systems central to the species' range. Despite evidence of connectivity, estimates of nucleotide diversity and effective population size were low across all populations, suggesting the patchy distribution of *F. latifolia* populations may impact its long‐term evolutionary potential. Range‐wide estimates of genomic offset, which indicate genomic change required to adjust to future climate projections, were greatest in the eastern and lowest in the southern portions of the species' range, suggesting the regional distribution of genomic variation may impact evolutionary potential longer‐term. To preserve evolutionary capacity across populations needed for the development of breeding and restoration programmes, prioritising conservation of range‐wide genomic diversity will provide a foundation for long‐term species management.

## 1 Introduction

The International Union for Conservation of Nature (IUCN) recently identified the maintenance of intraspecific genomic variation for species of conservation concern as a ‘hidden biodiversity crisis’ (IUCN Standards and Petitions Committee, 2022; reviewed in Des Roches et al. 2021; Hoban et al. 2021). As the raw material upon which natural selection acts, maintenance of genomic variation is critical to maintaining evolutionary potential (reviewed in Kardos et al. 2021; Mclaughlin et al. 2025). The combination of local extinctions, demographic declines and selection in response to anthropogenic influences is threatening the persistence of intraspecific population genomic variation globally (Des Roches et al. 2021). To preserve long‐term evolutionary potential for species at risk, understanding the evolutionary processes contributing to standing genomic variation is necessary. Landscape genomic analyses can inform conservation and restoration by assessing species‐wide diversity and differentiation (Laporte and Charlesworth 2002; Vandergast et al. 2007; Nock et al. 2011; Li et al. 2019; Osuna‐Mascaró et al. 2023; reviewed in Savolainen, Pyhäjärvi, and Knürr 2007; Hoban et al. 2016), estimating contemporary and predicting future genotype‐environment relationships (Meek et al. 2023; St. Clair et al. 2022) and evaluating effective population size (reviewed in Waples 2010) and relatedness (Félix‐Valdez et al. 2016; Tabas‐Madrid et al. 2018; Ruiz Mondragón et al. 2023). Such analyses can be invaluable to informing proactive conservation strategies, including the expansion of *ex situ* collections and establishment of genecological resources that can be foundational to restoring species at risk (Cavender et al. 2015; Di Santo et al. 2022; Kardos et al. 2018; Diaz‐Martin et al. 2023; Kleinman‐Ruiz et al. 2017; Kleinman‐Ruiz et al. 2022; reviewed in VanWallendael, Lowry, and Hamilton 2022).

As a keystone tree species, ash (*Fraxinus* L.; Oleaceae) species provide essential ecosystem services, including carbon sequestration, nutrient and water cycling across North American forests (Barstow et al. 2018; Gandhi and Herms 2010; Hausman, Jaeger, and Rocha 2010; Herms and McCullough 2014). However, the introduction of the emerald ash borer (EAB; *Agrilus planipennis* Fairmaire; Buprestidae) has decimated *Fraxinus* species across much of eastern North America, causing one of the costliest forest insect invasions to date across the United States (Kovacs et al. 2010; Semizer‐Cuming et al. 2019). With the continued range expansion of EAB, officially observed in Oregon, USA in 2022 and British Columbia, Canada in 2024, efforts to conserve and restore *Fraxinus* species have emphasised *ex situ* collections to preserve standing genomic variation and germplasm maintenance to lay a foundation for EAB‐resistance breeding and future restoration (Carlson, Cunningham, and Westley 2014; Hamilton et al. 2020). Genomics‐driven monitoring of extant populations may be leveraged to understand the demographic history of the species and how different neutral and non‐neutral evolutionary processes have shaped standing genomic variation (Di Santo et al. 2022; Bolte et al. 2024). In addition, genotype‐environment associations (GEA) enable characterisation of the role of environmental adaptation in shaping genomic structure (reviewed in Wang and Bradburd 2014; Lasky, Josephs, and Morris 2023). Modelling the relationship between genotypic and environmental variation provides the opportunity to evaluate the impact environmental change may have to genotype‐environment associations, quantifying potential genomic mismatches under future climate conditions (i.e., genomic offset; Jia et al. 2020; Borrell et al. 2020; Mahony et al. 2020; Griffith et al. 2021; Láruson et al. 2022; St. Clair et al. 2022; Varas‐Myrik et al. 2022; Lind et al. 2024; reviewed in Capblancq et al. 2020). Such analyses could play a key role in restoration efforts by identifying populations or regions to be prioritised for *ex situ* collections, quantifying regions of connectivity to monitor for potential EAB movement, preserving genomic variation critical to adaptation under current and future environments and designing pest resistance breeding programmes.

Here, we focus specifically on *Fraxinus latifolia* Benth. (Oregon ash), a dioecious ash species characteristic of riparian areas across much of the Pacific Northwest of North America. With a geographic distribution ranging from southern California, USA to British Columbia, Canada, it is one of the few deciduous tree species widespread across western coastal forests. It plays critical roles in regulation of light and water regimes in the region and is commonly used in restoration, particularly within wetland systems where it plays a primary successional role (Prive 2016). While widespread, the impending threat and magnitude of mortality associated with EAB have garnered the species near‐threatened conservation status globally according to the IUCN Red List of Threatened Species (Oldfield and Westwood 2017). To date, no studies have been conducted describing the genomic structure of *F. latifolia* to ensure population genomic variation and adaptive evolutionary potential are considered for conservation and restoration management. *Fraxinus latifolia* provides an ideal case to evaluate factors influencing range‐wide patterns of genomic variation to support proactive conservation applications to expand *ex situ* collections, establish resources foundational to an EAB‐resistance breeding programme, and inform assisted gene flow for restoration plantings.

Leveraging the power of landscape genomics for a widespread species at risk enables identification of regions and populations of greatest conservation concern and can improve selection of populations for expanding *ex situ* collections and breeding programmes, to ultimately better target funding and effort across a large geographic range. While extensive preparation and response efforts (Bliss‐Ketchum et al. 2021) have been in place prior to EAB's detection in Oregon, USA and British Columbia, Canada, this study represents the first application of genomic data to inform ongoing and future conservation and restoration for *F. latifolia* . Using high‐throughput sequencing across populations spanning the distribution of *F. latifolia*, we will (i) characterise standing genomic variation within and among populations of *F. latifolia* , (ii) evaluate gene flow and identify potential corridors of connectivity across populations, (iii) assess genotype‐environment associations and predict capacity to adapt to change and (iv) identify populations or regions of greatest conservation concern. These data and analyses will provide a valuable reference for long‐term genomic monitoring across the species' range that will be critical as EAB continues to expand its range, and a baseline understanding of connectivity and adaptation needed to identify germplasm to target for current and future conservation, preservation and restoration efforts.

## 2 Materials and Methods

### 2.1 Population Sampling and DNA Extraction

**FIGURE 1:**
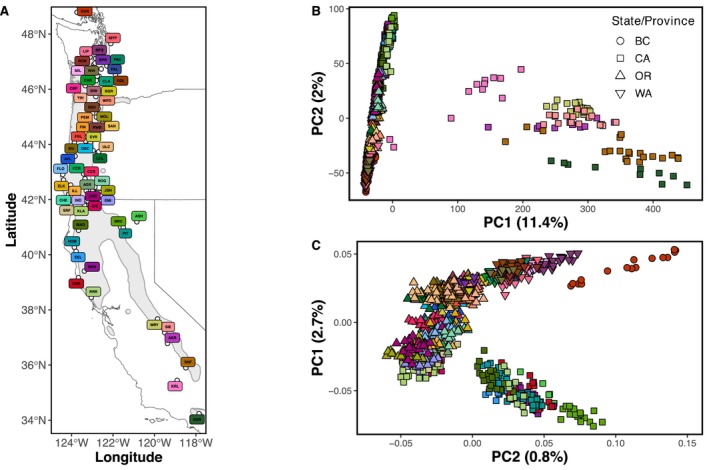
Map of sampled populations (A), PCA results for SNPs from all samples (B), and PCA results for all samples excluding southern populations (C). The southern samples were significant outliers in both the SNP PCA (B) and allelic proportions PCA (Figure S1) and were thus excluded from a second SNP PCA (C) and all subsequent analyses. The *x*‐axis of (C) is PC2, versus PC1, to highlight the geographic structure of the populations and match the orientation of the map, with the northernmost population being high on the y‐axis, and the southernmost populations being low. Colour of population markers in (A) is consistent across all figures and listed in Table S1. The grey outline and shaded region in (A) represent the range of *Fraxinus latifolia* , per Little (1971) and acquired from databasin.org.

Between the autumn of 2021 and spring of 2022, leaf tissue was collected from individuals spanning nearly the entire natural distribution of *Fraxinus latifolia* (Oregon ash; Figure 1A; Table S1). 61 populations were sampled across the species' distribution (California, Oregon, Washington and British Columbia) representing nearly the entire range of environments that *F. latifolia* occurs in for a total of 1083 individuals. When possible, at least 20 individuals were sampled per population. Due to habitat fragmentation across parts of the distribution, some populations had fewer than 20 individuals sampled. The number of individuals genotyped per population ranged from four individuals to 26 individuals (average individuals sampled per populations = 17.7, SD = 5.0). Fresh leaf tissue was dried on silica gel, and approximately 20 mg of dry tissue was used for DNA extraction. DNA was extracted locally using a Macherey‐Nagel NucleoSpin Plant 2 extraction protocol or outsourced to Ag‐Biotech (Monterey, CA, USA) for extractions via a high‐throughput modified CTAB protocol due to the large sample numbers. The concentration and purity of extracted DNA were quantified for each sample using a NanoDrop 1000 Spectrophotometer (Thermo Fisher Scientific; Waltham, MA, USA) to ensure all samples had a concentration of at least 2.0 ng/μL and purity ratios, per 260/280, of 1.21 to 2.09 (average 260/280 = 1.93).

### 2.2 Genomic Library Preparation and ddRADseq

Genomic libraries were prepared using a double‐digest restriction‐site‐associated DNA sequencing (ddRADseq) protocol per Parchman et al. (2012) at the University of Nevada—Reno. Genomic DNA was digested using *Eco*RI, a methylation‐sensitive restriction enzyme, and *Mse*I, a methylation‐insensitive restriction enzyme, endonucleases (New England BioLabs Inc). The use of a methylation‐sensitive enzyme such as *Eco*RI will reduce the amount of repetitive, non‐coding DNA that is amplified and allow for greater reproducibility among future datasets generated to monitor for potential changes in genomic diversity under risk of EAB invasion among *F. latifolia* populations. T4 DNA ligase (New England BioLabs Inc) was then used to ligate uniquely barcoded Illumina adaptors (576 unique barcodes, each matching a sample) to the *Eco*RI cut sites of fragments, and standard Illumina adaptors to the *Mse*I cut sites. Barcoded fragments were amplified via PCR using Iproof DNA polymerase (BioRad; Hercules, CA USA). PCR‐amplified genomic libraries were pooled and sent to the Genomic Sequencing and Analysis Facility (GSAF; Austin, TX, USA) for size selection of fragments and sequencing. Size fragments within the range of 350–450 bp were selected using a Pippin Prep quantitative electrophoresis unit (Sage Science; Beverly, MA, USA). Single‐end sequencing of 100 bp read lengths was performed on two lanes of an Illumina NovaSeq 6000 platform using S2 chemistry.

### 2.3 De Novo Assembly and SNP Calling

To detect and discard potential contaminants, such as *Escherichia coli* , *PhiX* or Illumina‐associated oligonucleotides, we first used the ‘Tapioca’ pipeline (http://github.com/ncgr/tapioca). Demultiplexing of sequence files by individual was performed using a custom Perl script, which was also used to correct barcode sequencing errors, trim cut sites and barcoded oligo‐associated bases. Reads were filtered and assembled *de novo* using ‘cd‐hit‐est’ (Fu et al. 2012) following procedure detailed in ‘dDocent’ v2.7.8 (cutoffs: individual = 8, coverage = 6; clustering similarity: ‐c = 0.96; Puritz, Hollenbeck, and Gold 2014). This step generated a set of contig consensus sequences representing a reference of genomic regions sampled by the reduced representation sequencing data. Demultiplexed reads were mapped to the reference assembly using ‘bwa‐mem’ v0.7.17 using default parameters (Li 2013). Sequence variants were identified using ‘bcftools’ v1.9 (Danecek et al. 2021) and subsequent filtering was completed using ‘vcftools’ v0.1.16 (Danecek et al. 2011). Biallelic single nucleotide polymorphisms (SNPs) present within at least 90% of the sequenced reads across all samples were retained. These SNPs were further filtered to one per reference contig to reduce effects of linkage disequilibrium. Additional filtering included cut‐offs of minor allele frequency ≥ 2%, mean read depth across samples ≥ 5 and < 15, and alternate allele call quality ≥ 999. SNPs with excessive coverage (i.e., with a mean read depth of > 15) were removed and only those with two alleles present were retained to ameliorate potential genotyping bias from mis‐assembly of paralogous genomic regions (Hapke and Thiele 2016; McKinney et al. 2018). As mis‐assembly of paralogous genomic regions can lead to an abnormal excess of heterozygosity, SNP‐specific *F*_IS_ was calculated, and only SNPs with *F*_IS_ > −0.5 were retained (Hohenlohe et al. 2013; McKinney et al. 2017). Individuals missing more than 40% of identified SNPs were removed from subsequent analyses (*n* = 30) resulting in a total of 998 individuals sequenced across 42,759 SNPs for inclusion in analyses below.

### 2.4 Analysis of Population Genomic Structure and Diversity

To assess range‐wide genomic structure of *F. latifolia*, a PCA was performed including all populations using the *snpgdsPCA* function within the ‘SNPRelate’ v1.34.1 R package (Zheng et al. 2012). In the southern portion of the *F. latifolia* distribution, hybridisation with *F. velutina* Torr. has been reported and could involve production of tetraploids (Taylor 1945; Munz and Laudermilk 1949; Twisselmann 1967). Thus, to assess the potential for polyploidy, ploidy level variation was evaluated using the ‘gbs2ploidy’ v1.0 R package (Gompert and Mock 2017) in R version 4.4.0 (R Core Team 2024). See Appendix S1 for more details. Samples from the southern terminus of the range (six populations, 92 individuals) were identified as outliers in principal component space, indicating substantial genomic differentiation in addition to evidence of polyploidy (see Appendix S1 for more details). Thus, all downstream analyses were performed excluding these individuals. To further characterise genomic structure and estimate admixture coefficients, a *structure* analysis was performed using ‘fastStructure’ (Raj, Stephens, and Pritchard 2014) as implemented in the ‘structure‐threader’ suite (Pina‐Martins et al. 2017). The best‐supported *k* ‐value was used to assess the number of genomic clusters (*k* = 2–6 were selected to test more potential *k* values than would be predicted by PCA results—See Results—Population Genomic Structure for more detail) using the greatest marginal likelihood test as implemented by the ‘chooseK.py’ script from ‘fastStructure’ based on the remaining 906 individuals sampled across the species' distribution.

Nucleotide diversity (*π*; Nei and Li 1979) and Watterson's *θ* (Watterson 1975) were calculated to describe population‐specific variation in the distribution of polymorphisms. In addition, Tajima's *D* (Tajima 1989) was used to evaluate the mean number of pairwise differences across segregating sites for each population. This approach uses the distribution of rare alleles to indicate where and how different selective and demographic events may have shaped genomic variation. Analyses were implemented in ‘ANGSD’ v0.923 (Korneliussen et al. 2013; Korneliussen, Albrechtsen, and Nielsen 2014) using methods that incorporate genotype uncertainty as this limits the potential impact of errors associated with mis‐called genotypes where using low‐coverage sequence data (Korneliussen et al. 2013). The folded site allele frequency likelihoods were estimated with *realsfs*, which calculated genotype likelihoods using the setting ‘GL 1’ estimated from the model implemented in ‘samtools’ to obtain the likelihood of the folded site frequency spectrum (SFS). Nucleotide diversity was estimated using *doThetas 1* and *thetastat* commands using SFS likelihoods as priors, respectively, for each SNP across the reference assembly and averaged the measures for each population.

To estimate levels of inbreeding within populations, inbreeding coefficients (*F*_IS_) were calculated using the *gl.report.heterozygosity* function in the R package ‘dartR’ v2.9.7 (Mijangos et al. 2022) and relatedness within populations, quantified via identity‐by‐descent, was evaluated using the R package ‘SNPRelate’ v1.34.1′ (Zheng et al. 2012). Using the *snpgdsIBDMoM* and *snpgdsIBDMLE* functions, which use method of moments (MoM) (Purcell et al. 2007) and maximum likelihood estimation (MLE) (Milligan 2003; Choi, Wijsman, and Weir 2009) methods, respectively, kinship coefficients for each possible pair within a population were compared. Finally, to estimate contemporary effective population size (*N*_e_), the linkage disequilibrium effective population size (LD‐N_e_) was estimated for each population using the *ldNe* function from the ‘StrataG’ R package v2.5.01 (Archer, Adams, and Schneiders 2017) per methods described by Waples, Larson, and Waples (2016), with a minimum allele frequency of 0.05 and default parameters. Effective population size provides a valuable population parameter related to genetic drift, predicts the subsequent rate of loss of heterozygosity over generations due to a finite population size, and can relate to levels of the increase in homozygosity of identical by descent alleles due to inbreeding (Franklin, Soulé, and Wilcox 1980; Braasch et al. 2021; reviewed in Waples 2022, 2024; Gargiulo et al. 2024).

To quantify genomic differences among populations, SNP‐specific pairwise estimates of Hudson's *F*_ST_ (Hudson, Slatkin, and Maddison 1992) and Nei's *D* (Nei 1972) were calculated from allele frequencies. Hudson's *F*_ST_ estimation was selected as it is consistent across sampling schemes and independent of sample composition (Bhatia et al. 2013). Along with using an *F*_ST_ estimation that would be robust to variation in sample sizes across the populations, using sufficiently large SNP data sets (e.g., > 1500 SNPs) can provide accurate estimates of *F*_ST_ where samples are limited (Nazareno et al. 2017).

### 2.5 Estimates of Connectivity for *F. latifolia*

To quantify and visualise population connectivity across the range of *F. latifolia* , Estimated Effective Migration Surfaces (‘EEMS’; Petkova et al. 2016) were used to estimate species' effective migration surfaces. The ‘Plink’ v2.0 (Chang et al. 2015) function ‘‐‐make‐bed’ was used to convert the processed VCF to a BED file, allowing for non‐human chromosome counts with the ‘‐‐allow‐extra‐chr’ flag. A difference matrix using the ‘mean allele frequency’ imputation method was generated using the bed2diffs_v2 function from the ‘EEMS’ R scripts (https://github.com/dipetkov/eems; Petkova et al. 2016). Multiple ‘EEMS’ runs were performed to evaluate the goodness of fit of models derived using different ‘nDemes’ parameters. Models were developed using ‘nDemes’ starting at 100 and increasing by 100 each run until AICc scores no longer improved and observed demes did not increase. The best‐performing model was then selected using AICc scores. Models were generated using 25 million MCMC iterations, a burn‐in of 10 million iterations, and 9999 removed between samplings. The best model was then visualised across the landscape using the predicted migration rate (*m*; log10 scale).

In addition, expected heterozygosity (*H*_E_) and observed heterozygosity (*H*_O_) were calculated using the *gl.report.heterozygosity* function in the R package ‘dartR’ v2.9.7 (Mijangos et al. 2022). *H*_*O*_ was interpolated across the species' range to identify regions across the landscape that may be targets for additional *ex situ* collections. Interpolations were performed using the inverse distance weighting (IDW) method as implemented in the R package ‘spatstat’ v3.0‐3 (Baddeley, Rubak, and Turner 2015).

### 2.6 Quantifying the Influence of Geography and Environment on Genomic Variation

The multivariate relationship between geography, environment and genomic variation was quantified using 23 climatic variables averaged between 1991 and 2020 (Table S1) extracted from ClimateNA v7.41 (Wang et al. 2016) at a 1 km^2^ resolution based on longitude and latitude of population origins (Table S1). Elevation for each population was extracted using the ‘elevatr’ R package v0.4.5 (Hollister et al. 2023). Collinearity across climatic variables was assessed using the *cor* function in the base R package ‘stats’ to calculate Pearson correlations among climatic variables to limit potential redundancies. One variable from a pair with a correlation coefficient greater than |0.75| was excluded from further analyses. Seven climatic variables were retained following multicollinearity reduction, including frost‐free period (FFP), mean annual precipitation (MAP), mean annual temperature (MAT), precipitation as snow (PAS), relative humidity (RH) and continentality (TD, temperature difference between mean warmest month temperature and mean coldest month temperature; Table S1).

Both isolation‐by‐distance (i.e., IBD; population genomic distances associated with geographic distances) and isolation‐by‐environment (i.e., IBE; accumulation of genomic differences between populations from distinct environments), were evaluated using partial mantel tests with the *mantel.partial* function from the R package ‘vegan’ v2.6‐4 (Oksanen et al. 2022). The tests were performed using the Nei's *D* genetic distance metric, Euclidean environmental distances per the *dist* function of base R, Haversine geographic distances converted to kilometres per the *distHaversine* function of the ‘geosphere’ v1.5‐18 R package (Hijmans 2022), 999 permutations and Pearson's product–moment correlation. A Mantel test was also used to test for correlation between geographic and environmental distances.

To test for genotype‐environment associations and the impact of climate on population genomic structure, a redundancy analysis (RDA) was performed, with latitude and longitude included to represent population geographic structure (see Results). Prior to analysis, elevation and climate variables were scaled using the base R *scale* function, and SNP data were converted to minor allele counts using ‘vcftools’ (‐‐012 output format). The *rda* function from the R package ‘vegan’ v2.6‐4 was used to perform RDA using uncorrelated environmental variables, including latitude and longitude to represent geography, and elevation, frost‐free period (FFP; days), mean annual precipitation (MAP; mm), mean annual temperature (MAT;°C), relative humidity (RH; %) and continentality (TD;°C) to represent the environment. The ‘vegan’ v2.6‐4 function *varpart* was used to perform a variance partitioning analysis based on the RDA model to evaluate geographic distance, environmental distance, and their individual and combined effect on genomic distance among populations.

### 2.7 Predicting Genomic Offset Under Climate Change

A generalised dissimilarity model (GDM) was generated to model changes in genomic distances along climatic gradients and forecast genomic changes required to maintain genotype‐environment relationships under future climate conditions (i.e., genomic offsets; Fitzpatrick and Keller 2015). Genomic distances were calculated using the *dist.genpop* function of the ‘adegenet’ R package (Jombárt 2008) for all SNPs, and climatic variables previously used for RDA and IBE were used as the environmental matrix (FFP, MAP, MAT, PAS, RH and TD). The *gdm* function within the R package ‘gdm’ v1.5.0‐9.1 (Fitzpatrick et al. 2022) was used to fit the generalised dissimilarity model to the genomic distance and climatic data. Ensemble projections associated with future climate averages between the years 2041 and 2070 using three climate change emission scenarios: ssp245 (a less severe change in climate assuming a decrease in carbon output), ssp370 (a very likely scenario with continued carbon output) and ssp585 (a ‘worst case scenario’ model assuming an increase in carbon output; Mahony et al. 2022) were used for GDM. Ensemble models were used to account for potential spatio‐temporal variation associated with individual models to increase confidence in climate change projections (Mahony et al. 2022; reviewed in Hausfather et al. 2022). The *gdm.transform* function was used to rescale climatic variables based on variable importance in the GDM. Transformed climatic variables were then used as input for a PCA using the base R prcomp function with a random sampling of 50,000 raster cells. The first three principal components were mapped to RGB colour values which were combined for each cell and projected onto the landscape to evaluate how future climate projections will contribute to genomic offset across the species range.

## 3 Results

### 3.1 Population Genomic Structure

A total of 245 Gb of sequence data across 2.85 billion reads were generated. The average read count per sample was 2.63 × 10^6^ ± 1.44 × 10^6^ with a mean quality score of 36 and GC% of 40. A total of 2.82 × 10^9^ reads had a quality score of 30 or above. After mapping and variant calling, we initially retained 1,280,333 biallelic SNPs across the 1083 individuals. Following stringent filtering, 42,759 SNPs remained for 998 individuals (average sequencing depth = 7.92×) representing 61 populations across the range of *F. latifolia* (Figure 1A; Table S1 for population sampling information).

An initial principal components analysis using genomic data for all individuals revealed two distinct clusters largely differentiating the northern and southern distribution of sampled populations. PC1 explained 10.34% of the variance distinguishing two distinct clusters and PC2 1.96% of the variance (Figure 1B; Figure S1B). One cluster comprised individuals sampled from British Columbia, Washington, Oregon, and the northern coast of California, while the second cluster included individuals sampled from the southern range of the Sierra Nevada mountains and disjunct southwestern populations. Given the gap in sampling between the northern and extreme southern groups, uncertainty in the potential for hybridisation between *F. latifolia* and *F. velutina* in the south, and evidence here (Figure S2) and elsewhere (Taylor 1945; Munz and Laudermilk 1949; Twisselmann 1967) for polyploidy in the southern part of the range, we limited subsequent landscape genomic analyses to 906 samples from 55 populations (~85% of our samples), excluding these six genomically distinct populations comprising the southern populations cluster (Appendix S1, Table S2). Subsequent analysis based on the *F. latifolia* populations ranging from British Columbia to northern California identified three clusters that largely followed a north‐to‐south latitudinal gradient indicated by a strong correlation between latitude and PC1 (*r* = 0.931; Figure 1C).

**FIGURE 2:**
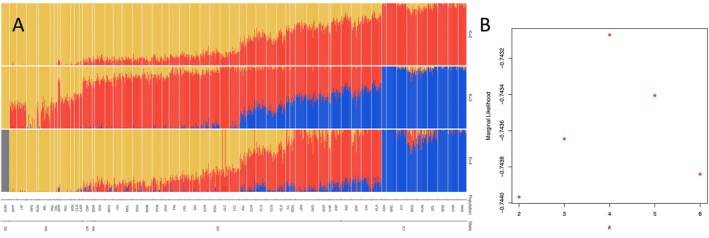
Visualisation of ancestry Q‐scores from *Structure* analysis (A) and marginal likelihood scores for *k*'s two through six (B). A north‐to‐south pattern of clustering is identified for all clusters, similar to PCA results. In *k* = 4, the British Columbia, Canada population forms a distinct cluster (dark red), all Washington, USA and northern Oregon, USA populations from the Columbia River watershed form a cluster (yellow), southern Oregon and northern California populations from the Klamath‐Siskiyou Ecoregion form a cluster (red), and all other California populations form a cluster (blue). A *k* of two had the lowest marginal likelihood score and a *k* of four had the highest (B).

*Structure* analysis identified *k* = 4 (marginal likelihood = −0.743; Figure 2; Figure S3) as the model that maximised marginal likelihoods of cluster assignment and *k* = 2 (marginal likelihood = −0.744; Figure 2) as the number of clusters that best explain genomic structure within the data. For *k* = 2, a north‐to‐south gradient was evident, with the British Columbia, Canada population comprising a ‘northern’ cluster, and samples exhibiting mixed ancestry across clusters within northern Oregon along the Willamette River Valley differentiated from southern populations of the southern cluster in southern Oregon and California (Figures 2A and S3). The north‐to‐south gradient was prominent at *k* = 4, with clusters representing (i) the British Columbia, Canada population, (ii) Washington to central Oregon, USA populations, (iii) southwest Oregon and northwest California, USA populations adjacent to Oregon, (iv) and all other California, USA populations (Figures 2A and S3).

### 3.2 Population Genomic Diversity Analyses

**FIGURE 3:**
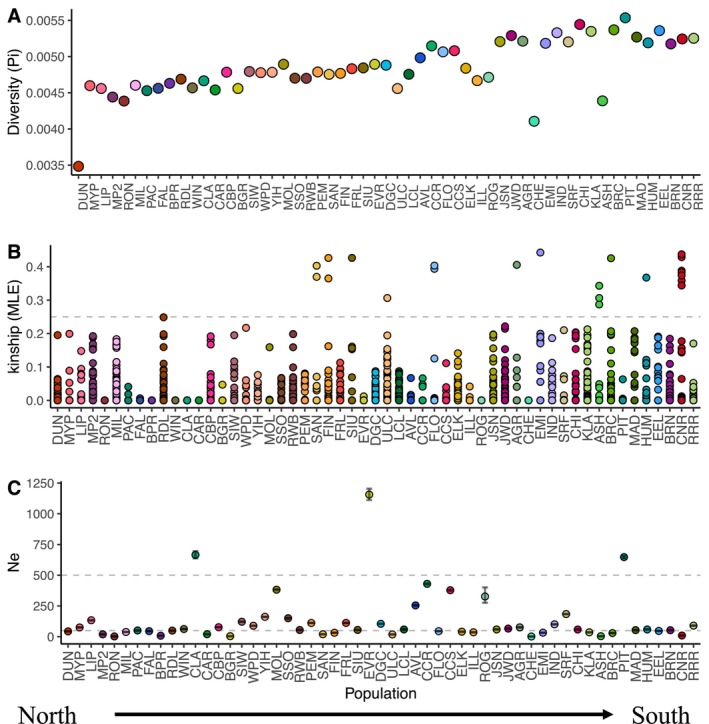
Dotplots visualising nucleotide diversity (π; A), MLE relatedness estimates (B) and LD‐N_e_ (C), per population, ordered by decreasing latitude. π estimates were generally very low across populations ranging from 3.48 × 10^−3^ and decreased with increases in latitude. Relatedness estimates were low, with means per population of MLE estimates ranging from 0 to 0.03, with similar results for MoM estimates. Horizontal lines in (B) designate the coefficient of full‐sibs, 0.25. Points above these lines represent samples with kinship coefficients greater than 0.25 and are more closely related than full‐sibs. Effective population size estimates (C) were very low for all populations, with all but three falling below 500 (PIT = 650, CLA = 670 and EVR = 1160). Horizontal lines are at 50 and 500, which are commonly accepted thresholds for *N*_e_ to combat inbreeding depression (50) and genetic drift (500). Error bars represent upper and lower limits of 95% confidence intervals for LD‐N_e_ estimation for each population.

Genome‐wide estimates of nucleotide diversity (*π*) were low across all populations. The northernmost population in British Columbia (DUN) exhibited the lowest nucleotide diversity (3.48 × 10^−3^ ± 1.49 × 10^−5^). Populations at the northern limits and in smaller river valleys towards the eastern and western range extents exhibited reduced nucleotide diversity relative to those populations in the central river valleys of the species' distribution. Estimates of *π* ranged from 3.48 × 10^−3^ ± 1.49 × 10^−5^ (DUN, British Columbia, Canada) to 5.54 × 10^−3^ ± 1.66 × 10^−5^ (PIT, California, USA), with a mean of 4.85 × 10^−3^ (Figures 3A and S4A). Similar to *π*, Watterson's *θ* values were low across all populations, exhibiting a similar latitudinal distribution, from 2.28 × 10^−5^ ± 8.96 × 10^−6^ (DUN, British Columbia, Canada) to 5.13 × 10^−3^ ± 1.23 × 10^−5^ (PIT, California, USA), with a mean of 4.20 × 10^−3^ (Figure S4B). Tajima's *D* values were negative for all but one population (CHE, Oregon, USA; Figure S4C), indicating a potential recent population expansion after a genetic bottleneck. Tajima's *D* ranged from −3.72 × 10^−2^ ± 4.83 × 10^−5^ (HUM, California, USA) to 1.91 × 10^−3^ ± 2.74 × 10^−5^ (CHE, Oregon, USA), with an average of −2.48 × 10^−2^.

Estimates of *F*_IS_ were generally low, ranging from −0.151 (DUN, British Columbia, Canada) to 0.056 (SRF, California, USA) with a mean of 0.014 ± 0.035, suggesting outbreeding within populations typical of a dioecious system. Identity‐by‐descent analysis also produced very low relatedness estimates. Only 0.3% (24 of 7810) of pairwise kinship coefficients were greater than 0.25. The majority of kinship coefficients (7786 of 7810 comparisons) were very low (kinship coefficient < 0.25). Mean MoM estimates of relatedness per population ranged from 0 (no identity‐by‐descent among compared samples; RON and WIN, Washington, USA) to 0.114 (DUN, British Columbia, Canada), with a mean estimate of 0.016. Mean MLE estimates of relatedness per population ranged from 0 at seven populations to 0.03 (ASH, California, USA), with a mean estimate of 6.09 × 10^−3^ (Figure 3B). Estimates of LD‐N_e_ were generally low, ranging from 1.45 (CHE, Oregon, USA; *n* = 4) to 1160 (EVR, Oregon, USA; *n* = 19), with a mean estimate of 128 ± 201 across all populations (Figure 3C; Table S3). Only three populations exceeded *N*_e_ = 500 (PIT = 650, CLA = 670 and EVR = 1160), and greater than 20 populations had *N*_e_ < 50, indicating limited evolutionary potential species‐wide.

Genomic differentiation among the populations was moderate with low *F*_ST_ and Nei's *D* values. *F*_ST_ values ranged from 0.030 (CCR, Oregon vs. CCS, Oregon) to 0.242 (CHE, Oregon, USA vs. DUN, British Columbia, Canada), with a mean of 0.078 (Figure S5) and Nei's *D* values ranging from 9.57 × 10^−3^ (CCR, Oregon, USA vs. CCS, Oregon, USA) to 0.094 (CHE, Oregon, USA vs. DUN, British Columbia, Canada), with a mean of 2.83 × 10^−2^ (Figure S5).

### 3.3 Estimates of Connectivity Across the Range of *F. latifolia*

**FIGURE 4:**
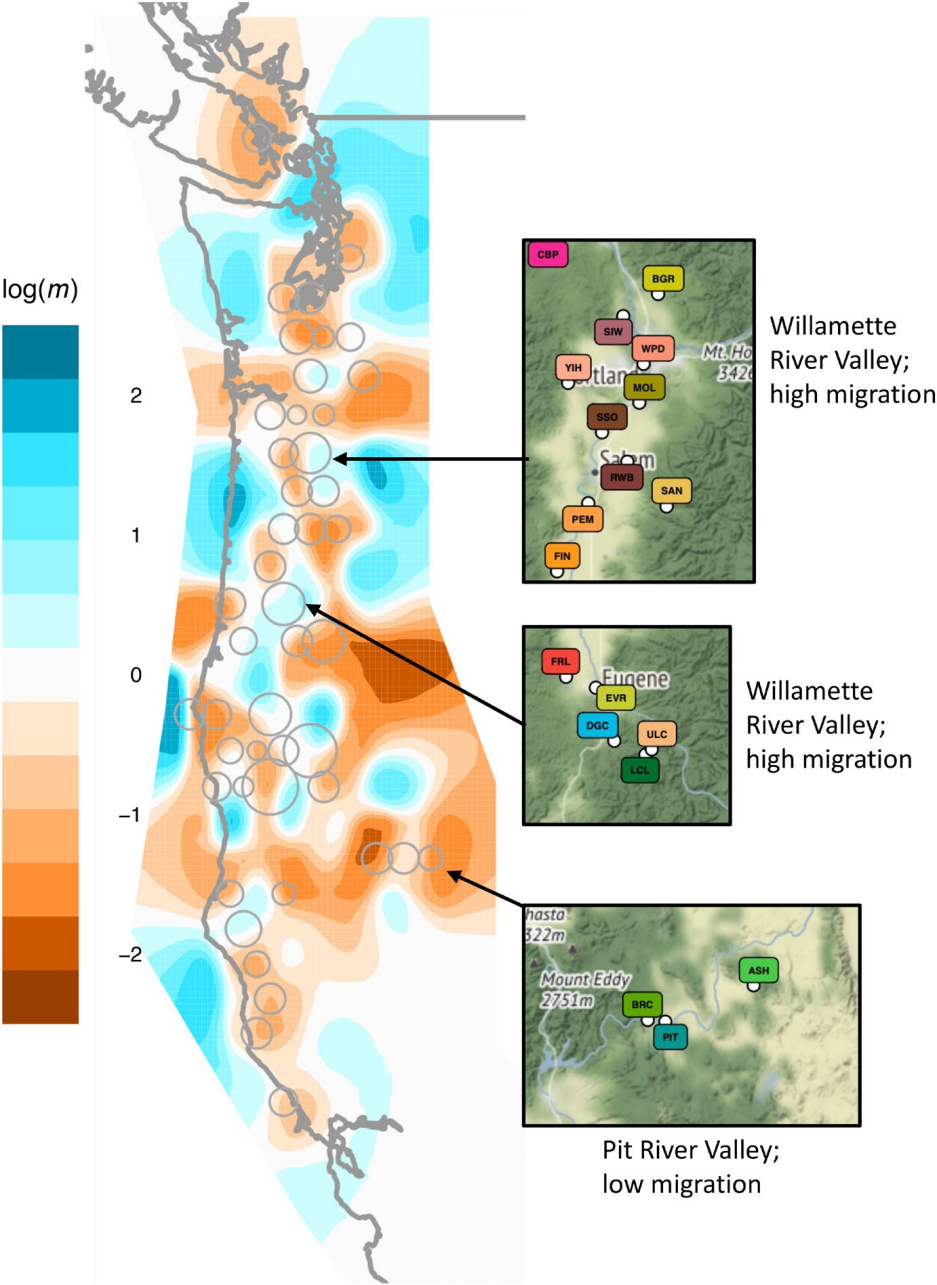
Estimated effective migration surface (‘EEMS’) visualisation for *F. latifolia* . Differences in migration rates (*m*; on a log10 scale) and deme connectivity that were significantly higher than the overall average rates are represented in blue (i.e., corridors of gene flow) and significantly lower than the overall average rate (i.e., barriers to gene flow) are represented in brown. Demes used in the estimation of the migration surface are represented by dark grey circles, with the size of the circle corresponding to the number of samples within the deme. Overall, deme connectivity, and thus effective migration, was greatest along major river systems, such as Willamette River in Oregon. River systems outside of the central valleys of the species range featured less connectivity and effective migration (e.g., the Pit River system).

‘EEMS’ was used to identify departures from isolation‐by‐distance (IBD) to detect potential barriers to gene flow or areas with greater rates of effective migration than expected given geographic distance and to visualise population connectivity over the range *of F. latifolia* (Figure 4). A total of seven ‘EEMS’ runs were performed, with ‘nDemes’ ranging from 100 to 700, as likelihood scores and observed demes per nDeme no longer improved at higher nDeme values. Per likelihood metrics, the fifth run, with a demes grid of 500 Demes, was the best‐fit model. Population connectivity, per estimated migration rates (*m*), was highest among demes occurring along major river systems in Oregon and northern California, indicating greater potential gene flow and genomic similarity than expected given their geographic distance. Estimates of effective migration rates were much lower outside of major river systems, indicating much higher genomic distances than expected given their geographic distances, suggesting the larger, more open riparian habitats serve as important corridors of gene flow for *F. latifolia* populations.

Expected heterozygosity, *H*_E_, and observed heterozygosity, *H*_O_, were estimated to evaluate genomic diversity across corridors of connectivity. Estimates of *H*_E_ ranged from 0.182 ± 0.278 (CHE, Oregon, USA) to 0.248 ± 0.194 (KLA, California, USA) with a mean of 0.220 ± 0.020. Estimates of *H*_O_ ranged from 0.143 ± 0.189 (CHE, Oregon, USA) to 0.244 ± 0.165 (CHI, California, USA) with a mean of 0.226 ± 0.014. Greatest interpolated *H*_O_ (Figure S6) was observed within populations occurring within large river systems central to the species' distribution and coincides with predicted corridors of gene flow estimated from ‘EEMS’ (Figure 4). Populations outside of the central valley systems, such as DUN, British Columbia, Canada and CHE, Oregon, USA, exhibited much lower *H*_O_. *H*_E_ and *H*_O_ were similar on average (mean *H*_O_/*H*_E_ = 0.974 ± 0.048), although regionally several populations had much lower observed than expected heterozygosity (*H*_O_/*H*_E_ < 0.9; CHE = 0.783, DUN = 0.837, ASH = 0.872 and BPR = 0.892) indicating that these populations may experience decreased gene flow, and could be more susceptible to drift.

### 3.4 Quantifying the Relationship Between Environment, Geography and Genomic Variance

**FIGURE 5:**
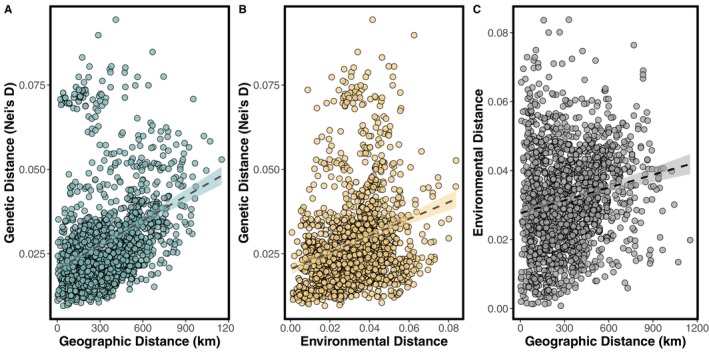
Results of IBD, IBE and IBD‐IBE analyses. Positive relationships were identified with Nei's *D* for both geographic distance (A; *r* = 0.337, *p*‐value = 0.001), environmental distance (B; *r* = 0.208, *p*‐value = 0.027), environmental distance by geographic distance (C; *r* = 0.182, *p*‐value = 0.004). Variance partitioning indicated that the combined effects of geography and environment explained 29.3% of the genomic variance among populations (residuals = 0.685; Figure 4D). Independently, geography and environment respectively accounted for 0.9% and 1.2% of genomic variance.

Variance partitioning indicated that the combined effects of geography and environment explained 29.3% of the genomic variance among populations (residuals = 0.685). Independently, geography and environment respectively accounted for 0.9% and 1.2% of genomic variance. Both IBD and IBE had statistically significant influences on genomic variation; however, their joint influence was greatest. Partial Mantel tests were statistically significant for IBD with IBE removed (*r* = 0.337, *p*‐value = 0.001; Figure 5A) and IBE with IBD removed (*r* = 0.208, *p*‐value = 0.027; Figure 5B), indicating that while both geography and environment influence population genomic structure of *F. latifolia* , geography alone explains more than environment. The Mantel test for environmental distance by geographic distance was also statistically significant (*r* = 0.182, *p*‐value = 0.004; Figure 5C).

**FIGURE 6:**
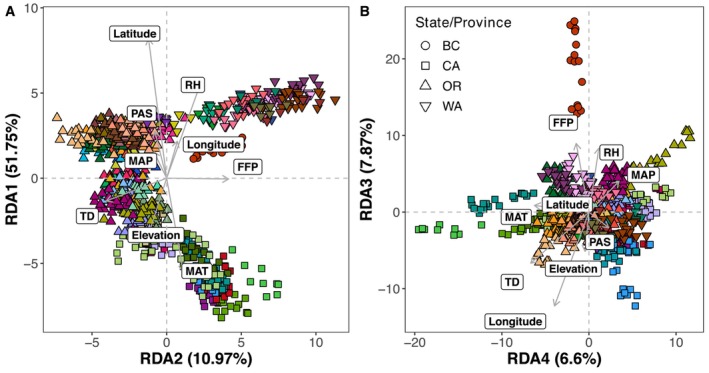
Population genomic structure was strongly associated with climatic variation across the range. Redundancy analysis (RDA) was performed to assess associations between geographic and climatic variables with genomic variation (i.e., SNPs). The direction and length of each arrow correspond with the strength of prediction (i.e., loading) along each RDA axis. RDA1, primarily comprising latitude and mean annual temperature (MAT), accounted for 51.75% of genomic variances explained by the RDA model. RDA2, comprising largely frost‐free period (FFP) and continentality (TD), explained 10.97% of genomic variance.

Environmental and distance variables that contribute to genomic structure were identified using RDA. RDA1 explained 51.75% of genomic variance (Figure 6), with latitude, mean annual temperature (MAT) and relative humidity (RH) having the highest predictive loadings (eigenvectors). RDA2 explained 10.97% of variance with frost‐free period (FFP) and continentality (TD; Figure 6B) having the greatest predictive loadings. RDA3 explained 7.87% of variance with longitude (Figure 6C) having the greatest predictive loading. Among northern populations (British Columbia and Washington) genomic structure was best explained by MAT (RDA1) and FFP (RDA2), while the regional genomic cluster including Oregon, and northern California populations was best explained by reduced MAT and greater RH (RDA1) and reduced continentality (RDA2). Southern California populations exhibited higher MAT on RDA1 and greater FFP (RDA2) relative to other regions. Overall, climatic gradients emphasise regional differences in genetic variance across the species' range that are likely partially shaped by selection.

### 3.5 Predicting Genomic Offset Under Climate Change

**FIGURE 7:**
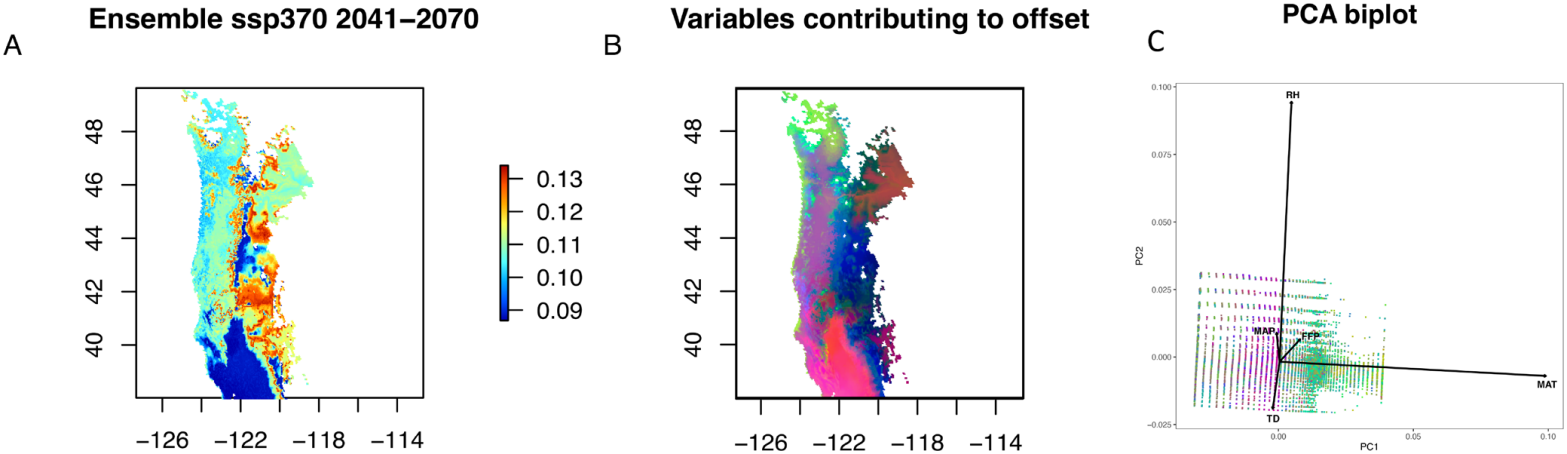
Visualisation of predicted genomic offset (A visualises predicted genomic offset under SSP370; other SSPs are visualised in Figure S7), principal components of climatic variables most greatly contributing to predicted genomic offset (B) and bi‐plot of principal components (C). Bi‐plot of PCA results (C) show the correlations between input variables and how they contribute to principal components. Variables that intersect at 90° angles are not correlated, variables that form very acute angles are positively correlated, and variables that form near 180° angles are negatively correlated. The length of the bi‐plot arrow shows the relative contribution to a given principal component. Predicted genomic offsets were consistent across all shared socio‐economic pathways (SSP) and ranged from 0.087 to 0.131 (mean = 0.106) for the most likely climate change scenario (ssp370; A). (B) Principal component 1 (red), primarily comprising MAT affects most of the range, principal component 2 (green), comprising largely RH, affect primarily the higher elevation areas along the primary valley systems within the species range and principal component 3 (blue), comprising all other climatic variables, largely affects the eastern part of the range.

A generalised dissimilarity model was used to model changes in genomic distances along climatic gradients and to predict ‘home vs. away’ genomic offsets (ranging from 0 to 1, with 0 being no predicted offset and 1 being all SNPs predicted to be maladaptive and populations become completely differentiated from one another at these loci), based on the uncorrelated climatic variables used in the RDA and variance partitioning analyses (FFP, MAP, MAT, PAS, RH and TD). The final GDM included five informative climatic variables: FFP, MAT, MAP, RH and TD. PAS was uninformative in GDM development. Evaluating different climate projection models indicated that ssp245, an optimistic scenario, exhibited predicted offset values from 0.087 to 0.130 (mean = 0.105), while ssp370, a very likely scenario exhibited values from 0.087 to 0.131 (mean = 0.106), and ssp585, a ‘worst case scenario’ exhibited values from 0.087 to 0.131 (mean = 0.108). Based on PCA, over much of the range of *F. latifolia*, predicted changes to mean annual temperature contributed to greatest genomic offset projections (Figures 7 and S7). However, in higher elevation regions, relative humidity had a stronger influence on genomic offset projections (Figure 7). All remaining climatic variables largely loaded on PC3, indicating greatest potential projected offset within the eastern portion of the species' range where fewer populations currently occur. The lowest projected offset was predicted in the southern portion of the species' range, particularly along the southwestern edges of the range, suggesting these populations may be ‘pre‐adapted’ to a warming climate and will experience reduced offset under global change. In contrast, northern and eastern regions had predicted genomic offsets of 0.1 or greater, indicating these populations may not have the genomic variation needed to respond to changing selective pressures.

## 4 Discussion

*Fraxinus latifolia* is foundational to wetland and riparian habitats of western North America. With the imminent threat of the emerald ash borer (EAB), understanding species‐wide standing genomic variation has become critical to designing effective conservation and pest‐resistant breeding programmes. The majority of standing genomic variation for *F. latifolia* is structured along a north‐to‐south climate and latitudinal gradient, with an ‘abundant center’ reflecting regions of nucleotide diversity and connectivity within central river valley systems and reductions in diversity among northern peripheral and disjunct populations in British Columbia, Oregon and California, respectively. Despite evidence of connectivity, however, effective population size is generally low across all populations, which may indicate dioecy and patchy distributions have impacted evolutionary potential. Given predictions associated with genomic offsets, this may indicate that genomic changes required to maintain genotype‐environment relationships under future forecasted conditions may be challenged and exacerbate risks associated with the invasion of EAB. However, these data provide an effective path forward for defining conservation units needed to mitigate both the risk of climate change and invasion by EAB, ultimately laying the foundation for a genomics‐informed conservation and EAB‐resistance breeding programme.

### 4.1 Population Genomic Structure Is Driven by Environment and Geography

The genomic structure of *F. latifolia* largely follows a latitudinal gradient (Figures 1C, 2 and S1C), with latitude a strong predictor of changes in allele frequencies. However, across the species' distribution, genomic substructure indicates the combination of different neutral and non‐neutral processes has likely influenced fine‐scale genomic structure. Populations sampled in Washington and Oregon, which occurred primarily along central valley river systems of the Columbia River watershed, formed a large cluster comprising much of the genomic diversity within the species (Figures 1 and 2). Other areas, such as southern California, which included populations within the Pit River valley from which more geographically isolated populations were sampled, formed distinct clusters (Figure 2). These results are consistent with an ‘abundant center’ model reflecting increased species abundance towards the centre of a distribution. Regions of greatest connectivity (Figures 4 and S5) and observed heterozygosity (Figure S6) were observed within the central portion of the species' range. This pattern is likely driven by the riparian nature of the species (Franklin and Dyrness 1973; Frenkel and Heinitz 1987), as the populations along the largest river valleys experience greater connectivity. Similar latitudinal gradients in the distribution of genomic variation have been observed for other coastal trees of the Pacific Northwest. These patterns have been attributed to range expansion during the warming early Holocene (~10,000 ybp) following the Pleistocene epoch (Pellatt, Hebda, and Mathewes 2001, reviewed in Jaramillo‐Correa et al. 2009), including *Picea sitchensis* (Bong.) Carr. (Mimura and Aitken 2007, 2010), *Pinus albicaulis* Engelm. (Liu et al. 2016), *Pinus monticola* Dougl. ex. D. Don (Rehfeldt, Hoff, and Steinhoff 1984; Richardson, Rehfeldt, and Kim 2009; Kim et al. 2011) and *Populus trichocarpa* Torr. & A.Grey ex. Hook. (Geraldes et al. 2014). Furthermore, distributions of nucleotide diversity associated with latitude suggest that post‐glacial expansion of *F. latifolia* likely followed from a single glacial refugia at the southern extent of the species' distribution, supporting the ‘leading edge hypothesis’ for post‐glacial expansion (Soltis et al. 1997).

Both geography and environment strongly influenced the distribution of genomic variation across the species' range. However, while both independently influence population genomic structure (Figure 5), their combined influence had the greatest effect (29.3% variance explained; Figure 5). This indicates that while both geographic and environmental distances do contribute significantly to population genomic structure, it is their combined influence that has the greatest predictive power in estimating genomic change.

### 4.2 Central River Valleys Serve as Corridors of Gene Flow

Population connectivity was greatest along major river systems within the centre of the species distribution and lower along peripheral river systems. *F*_ST_ was generally low (mean *F*_ST_ = 0.078 ± 0.030; Figure S5), indicating limited genomic differentiation among populations, with values in the range of those reported for other *Fraxinus* species (e.g., for *F. excelsior* L., Italian populations *F*_ST_ = 0.049 (Ferrazzini et al. 2016), British populations *F*_ST_ = 0.025 (Sutherland et al. 2010) and French populations *F*_ST_ = 0.016 (Sutherland et al. 2010)). However, the British Columbia, Canada (DUN) population (mean *F*_ST_ = 0.150 ± 0.024) and Chetco River, Oregon, USA (CHE) population (mean *F*_ST_ = 0.142 ± 0.025; Figure S5) were genomically differentiated from all other populations. The DUN population represents the northern limit of the species' range and is geographically isolated on Vancouver Island and disjunct from the mainland distribution (Figure 1A). ‘EEMS’ indicated that the Salish Sea likely acts as an effective barrier to migration and gene flow between the mainland and island populations (Figure 4). Similar patterns have been observed for populations of native tree species with island‐mainland distributions *Populus trichocarpa* (Geraldes et al. 2014) and *Pinus torreyana* Parry ex Carr. (Di Santo et al. 2022). The CHE population was sampled along the Chetco River in Oregon, USA. This river runs through rugged terrain that has likely acted as a physical barrier to gene flow, limiting potential influx of genomic variation from neighbouring populations. While *F*_ST_ values were generally low, EEMS predicted regional heterogeneity in migration. Estimated effective migration rates appear highest along major river systems within the central valleys of the species range, such as Willamette River in Oregon, USA and the Central Valley river systems of California (Figure 4), while peripheral river systems exhibited lower rates (Pit River and Russian River valleys in California; Figure 3). Regions with the greatest predicted effective migration reflect areas of greatest admixture also observed in the *structure* analysis (Figure 2). Populations from southern Oregon and northern California, which occur primarily along large river systems within the Klamath‐Siskiyou ecoregion that likely span a west‐to‐east region of connectivity (Eckert, Tearse, and Hall 2008) formed a distinct cluster. Those populations in the north and south that were more geographically isolated from riparian habitats outside of central river valleys also formed relatively discrete clusters (Figure 2). Climate change and other anthropogenic factors, such as urbanisation, could lead to greater fragmentation of habitat in which *F. latifolia* occurs, potentially leading to increased isolation among populations in the future.

The DUN population, geographically isolated on Vancouver Island, British Columbia, Canada was the most genomically distant of all populations (Figures 1, S4 and S5). This population comprised its own cluster within the *structure* analysis and was genomically differentiated from all populations (*F*_ST_ = 0.150 ± 0.024 and Nei's *D* = 0.058 ± 0.087; Figure S5), with extremely low nucleotide diversity (3.48 × 10^−3^ ± 1.49 × 10^−5^; Figure 3A). These results are likely attributed to past demographic changes, such as reduced connectivity, potential bottlenecks, and subsequent genetic drift following founding. These patterns are consistent with founding during the hypothesized northern expansion post‐glaciation, followed by a long period of isolation (Soltis et al. 1997). Fossilised *Fraxinus* pollen grains found in the Saanich Inlet of British Columbia, Canada date back 9–10 kybp (Pellatt, Hebda, and Mathewes 2001), suggesting this population likely was founded during this period, but has since become isolated and increasingly susceptible to loss of genomic variation through drift. Conservation efforts within British Columbia will help protect plants at the northern extreme of species range. Maintaining representatives from this population will ensure unique genomic variation is available for future use in breeding and restoration.

### 4.3 Small Effective Population Sizes in the Presence of Gene Flow

Estimates of effective population size (LD‐N_e_) were low across the range of *F. latifolia* (Figure 3C). Most populations exhibited values well below *N*_e_ < 500, with over twenty populations with *N*_e_ < 50, suggesting the evolutionary potential of *F. latifolia* populations has likely been negatively influenced by fine‐scale population substructure, drift, and a dioecious mating system. *Fraxinus latifolia* is a wind‐pollinated dioecious plant species, and previous studies have suggested that habitat fragmentation across male and female plants can impact genetic exchange, influencing population genomic structure and reducing estimates of *N*_e_ (Bacles et al. 2005; Eisen et al. 2023). Furthermore, the combined influence of landscape heterogeneity and genomic structure associated with latitudinal gradients suggests that fine‐scale population genomic substructure may have had a disproportionately negative influence on effective population size (Neel et al. 2013). Age stratification within populations has also been shown to negatively impact and bias effective population size estimates (Waples and Yokota 2007; Waples 2010; Waples, Antao, and Luikart 2014). *Fraxinus latifolia* is a long‐lived tree that generally does not reach reproductive maturity until approximately 30 years of age (Niemiec et al. 1995). However, while there is likely some degree of age stratification within our sampled populations, the majority of individuals were of reproductive age (Figure S8), reducing potential biases associated with age stratification. Finally, biased sex ratios could lead to increased levels of inbreeding within populations, reducing effective population size (Ellstrand and Elam 1993; Frankham 1995). Unfortunately, sex discrimination was not possible for all individuals, and therefore application of sex ratio estimates associated with *N*_e_ estimation methods could not be used to make inferences of effective population size. However, estimates of relatedness were extremely low within populations (Figure 3B), suggesting that *N*_e_ estimates were likely not biased by relatedness among individuals. Long periods of low *N*_e_ increase the risk of losing genomic diversity, leading to reduced adaptive capacity and limited ability to respond to stresses such as a changing climate or invasive pest (Reed and Frankham 2003).

### 4.4 Contemporary and Future Associations Between Genomic and Climatic Variation

In addition to strong associations with latitude, climatic gradients strongly influenced range‐wide population genomic structure for *F. latifolia* (Figure 5B; Figure 6). Significant isolation by environment suggested increased population genomic differentiation with increasing environmental distance (*r* = 0.208, *p*‐value = 0.027; Figure 5B). Of climate variables, mean annual temperature (MAT) had the greatest predictive power on average of genomic differences across the species' range (Figure 6). Regional genomic differences among more northern populations of British Columbia, Canada and Washington, United States, were best predicted by temperature‐related variables, including frost‐free period (FFP) and continentality (TD; Figure 6), distinguishing the cold and humid climates of northern British Columbia from the warmer, drier climates of California.

Genotype‐environmental relationships currently maintained across the range of *F. latifolia* may be impacted by projected future climatic conditions. Mean annual temperature (MAT) is predicted to change under all future climate change scenarios (IPCC 2022). Based on the RDA, MAT was significantly associated with population genomic structure for *F. latifolia* (Figure 6; Table S3). Evaluating genomic offset estimates, MAT was a primary predictor of future genomic offset across a large portion of the species' range (Figures 7 and S7). This suggests that as average temperatures rise across the range, populations will become more genomically distinct for genomic variation associated with adaptation to temperature gradients, even in areas with higher migration rates per EEMS (Figure 4). Thus, as local climates warm, changes in MAT will impact predicted genotype‐environmental associations, potentially leading to maladaptation in the novel climatic conditions. Furthermore, regional variation across the species' range suggests populations are differentially sensitive to projected changes in temperature (e.g., southern CA, USA populations are predicted to be less affected by increasing MAT than those in WA, USA). Similar patterns of increasing predicted genomic offsets at higher latitudes are seen in other western North American tree species, such as *Populus balsamifera* L. (Fitzpatrick and Keller 2015) and *Pseudotsuga menziesii var. glauca* (Mayr) Franco (Lind et al. 2024). Together, these data suggest that potential mismatches between standing genomic variation and the genomic variation needed to adapt to changing environmental conditions may have disproportionate effects across the species' range. However, these results should be interpreted with caution, as recent studies have shown that predicted genomic offsets may not necessarily represent ‘genomic vulnerability’ and may require experimental results from common gardens to confirm predicted effects of changing environments on a given genotype to inform restoration and assisted gene flow strategies (Lotterhos 2024).

### 4.5 Implications for Conservation

The invasive EAB has decimated native *Fraxinus* populations across eastern North America and has now been observed in Forest Grove, Oregon, USA in 2022 with indications that it is spreading locally and has been recently confirmed in British Columbia, Canada (April 2024). Where freezing temperatures have traditionally limited the spread of EAB, expected increases in temperature associated with climate change and increased frost‐free periods suggest that environmental barriers to invasion across the range of *F. latifolia* will likely be reduced (DeSantis et al. 2013). The increased risk of genomic offset posed by climate change, which may have fitness consequences associated with maladaptation to locally novel climatic conditions (Figures 7 and S7), puts *F. latifolia* at greater risk of local extirpations across its range. While estimates of population connectivity in the ‘abundant center’ along large river valleys central to the species' range, provide potential corridors of movement for pollen and seed flow, it is clear that these regions may also facilitate the spread of EAB due to increased connectivity and should be monitored.

Since 2019, efforts have been in place to conserve germplasm *ex situ* with a long‐term goal to assess resistance to EAB in *F. latifolia* populations leveraging knowledge from breeding programmes established in the eastern United States (Sniezko 2020; Bliss‐Ketchum et al. 2021). Through 2023, seeds from over 350 maternal parent trees have been collected and preserved at the USDA Forest Service's Dorena Genetic Resource Center (Cottage Grove, OR, USA) and the National Seed Laboratory (Dry Branch, GA, USA) providing a baseline of material to establish progeny trials and breed for resistance. While these collections will aid in establishing an EAB‐resistance breeding programme, the collections do not cover genomic variation spanning the range of *F. latifolia* . Given the results presented here, existing collections may lack genomic variation that represents neutral or adaptive genomic variation critical to long‐term species evolution. Thus, these data are invaluable to identifying regions to target additional *ex situ* collections for maintenance of germplasm needed for both progeny trials and long‐term conservation. Climate change will likely exacerbate the risks to *F. latifolia*, as the combination of abiotic and biotic threats has already led to high mortality in several *Fraxinus* species in eastern North America, Russia and Ukraine (reviewed in Sun et al. 2024). Conservation of genomic diversity will be key to the long‐term conservation and restoration of *F. latifolia* . Application of genomics to continued monitoring of genomic diversity, inbreeding and effective population sizes provides a proactive means to mitigate potential threats associated with an invasive species. An abundant centre of genomic diversity has been identified along central river valleys. Efforts to maintain standing genomic variation of this species should focus on the abundant centre and an expansion of *ex situ* collections from the four genomic clusters (British Columbia, Washington + northern Oregon, Southern Oregon + northern California and California). Ultimately, combining conservation and genomics approaches will facilitate breeding and screening for resistance by improving our ability to leverage standing genetic variation, creating a genomics‐driven pipeline for forest‐pest management critical to long‐term conservation, breeding and restoration.

## Author Contributions

R.S., T.T., W.W. and J.A.H. contributed to planning and performing sample collections. A.E.M., T.M.F., T.P. and J.A.H. designed the research. T.P. performed ddRADseq genomic library preparation and sequencing. A.E.M. and T.M.F. performed the analyses. A.E.M. wrote the initial draft of the manuscript. All authors contributed to the revision and finalisation of the manuscript.

## Conflicts of Interest

The authors declare no conflicts of interest.

## Supporting information


Appendix S1.

Table S1.

Table S2.

Table S3.

## Data Availability

Demultiplexed DNA sequences have been made available from the National Centre for Biotechnology Information database (BioProject: PRJNA1183741). Scripts used to perform dDocent and ANGSD analyses are available at https://github.com/trevorfaske/FRLA_analyses. Scripts to perform downstream analyses are available at https://github.com/aemelton/OregonAshLandscapeGenomics.
